# Cooperative Computing System for Heavy-Computation and Low-Latency Processing in Wireless Sensor Networks

**DOI:** 10.3390/s18061686

**Published:** 2018-05-24

**Authors:** Jongtack Jung, Woonghee Lee, Hwangnam Kim

**Affiliations:** School of Electrical Engineering, Korea University, Seoul 02841, Korea; skylover89@korea.ac.kr (J.J.); tgorevenge@korea.ac.kr (W.L.)

**Keywords:** wireless sensor networks, Internet of Things, cooperative computing, heavy computation, low-latency processing, audio data processing

## Abstract

Over the past decades, hardware and software technologies for wireless sensor networks (WSNs) have significantly progressed, and WSNs are widely used in various areas including Internet of Things (IoT). In general, existing WSNs are mainly used for applications that require delay-tolerance and low-computation due to the poor resources of traditional sensor nodes in WSNs. However, compared to the traditional sensor nodes, today’s devices for WSNs have more powerful resource. Thus, sensor nodes these days not only conduct sensing and transmitting data to servers but also are able to process many operations, so more diverse applications can be applied to WSNs. Especially, many applications using audio data have been proposed because audio is one of the most widely used data types, and many mobile devices already have a built-in microphone. However, many of the applications have a requirement that heavy-operations should be done by a tight deadline, so it is difficult for a single node in WSNs to run relatively heavy applications by itself. In this paper, to overcome this limitation of WSNs, we propose a new emerging system, *HeaLow*, a cooperative computing system for heavy-computation and low-latency processing in WSNs. We designed *HeaLow* and carried out the practical implementation on real devices. We confirmed the effectiveness of *HeaLow* through various experiments using the real devices and simulations. Using *HeaLow*, nodes in WSNs are able to perform heavy-computation processes while satisfying a completion time requirement.

## 1. Introduction

Supported by advancements of Micro Electro Mechanical Systems (MEMS), technologies of sensors have significantly progressed, and many various kinds of sensors have appeared [1]. With such progress, Wireless Sensor Networks (WSNs) have received a lot of attention from many researchers in various areas including Internet of Things (IoT) [2,3]. WSNs consist of sensor nodes which are connected with each other, and sensor nodes take the role of gathering sensed data and transmitting the data to users or servers [4,5,6]. In general, wireless sensor nodes have poor resources compared to wired nodes, because wired power sources are normally avoided for the mobility issue [7]. Therefore, existing WSNs are mainly used for delay-tolerant services that require low computation. For this reason, many researchers have focused on improving the power consumption efficiency of WSNs [8,9]. In addition, other researchers have conducted research on utilizing cloud servers to overcome the nodes’ limited resources such as low computing power, storage shortage, etc. [10,11,12,13,14]. This is because tasks with latency-tolerance or large scope aggregation property are performed well with the powerful resources of cloud servers [15].

Due to the advancement of hardware and software technologies in recent years, today’s mobile devices have more resources, sensors, and actuators. In addition to mobile devices, there have been significant developments on small-size and low-cost sensor platforms such as Arduino and WRTnode [16,17]. Using these devices, users are able to gather many different sensor data and perform heavy-computation required processing. Based on such advancement, the concept of edge computing was proposed. Edge computing refers to networks where edge nodes actively participate in computing operations [18]. Existing cloud computing models were mainly designed for traditional web applications, so those are not suitable for future Internet applications running on diverse mobile and sensor nodes [19]. Moreover, the network delay caused by long distances between cloud servers and devices is improper for latency-sensitive applications or services required to frequently transmit large-sized data such as video and audio data [16]. On the contrary, edge computing allows sensor nodes to properly utilize their resource to perform processing. Thus, wider range of applications can be run on sensor nodes with the edge computing concept.

As mentioned above, today’s nodes used for WSNs distinctly have more resources than those of the past. However, it is still hard for them to perform heavy-computation and low-latency processing. Thus, in addition to the edge computing, cooperative computing was proposed to utilize sensor nodes’ resources more efficiently [16,20]. In cooperative computing, sensor nodes share their resources, such as computing power and energy, and conduct processing cooperatively. The sensor nodes, based on cooperative computing, are able to offload the computation of processing sensed data to improve the computing performance and consume sensor nodes’ energy evenly. Thus, it is possible for nodes using cooperative computing to perform applications which require large computations. Especially, most applications using video data require processing a large amount of computations. Therefore, it is possible to improve the performance of such applications by applying the cooperative computing concept to the applications in WSNs. For instance, there is a research which proposed a system to use cooperative video processing to improve the quality of processing result [21].

In addition to video data, audio data are also among the most widely used data types in WSNs. A microphone is cheap and very widely used, so most mobile devices already have a built-in microphone. In addition, even though some sensor nodes do not have a built-in microphone, most of them are capable of using microphones. This means that applications using audio data are able to utilize nearby devices as sensor nodes easily. Because of these advantages, many researchers have been interested in utilizing audio data in WSNs, and various applications using audio data have been proposed [22,23,24,25,26]. To process or analyze the audio data in such applications, Fast Fourier Transform (FFT) [27] is almost always the key component. Even cross correlation [28] or wavelet transformation [29] is essentially a composition of multiple FFT operations or a partial result from FFT. Because FFT requires large computational resources, operations for audio data processing require a large amount of computation. Furthermore, many of the applications have a requirement that the operations should be done by the deadline, which makes it difficult for a single node in WSNs to run relatively heavy applications by itself.

To overcome the limitations, we propose a cooperative computing system, *HeaLow*, for heavy-computation and low-latency processing in WSNs. To finish the heavy-computation task by the deadline, *HeaLow* conducts processing and offloading properly by considering various factors. Because the audio data processing (acoustic signal processing) is one of the most widely used operations for applications in WSNs, we consider the audio data processing as the target task in this paper. However, *HeaLow* can be used for any applications which require large computations and processing to be completed within a tight deadline. The design goal of *HeaLow* is to aid the incapable wireless sensor nodes to finish heavy computations within deadline. In addition, if sufficient resources are present in the multi-device system, utilizing free resources to improve the application performance is the secondary goal of *HeaLow*. As timeliness of heavy computation is a challenging task, the power efficiency issue is not considered in this paper.

In this paper, taking advantage of the powerful resources of today’s devices, we focus on utilizing a few more powerful nodes for less powerful devices and propose the system enabling the incapable nodes to finish heavy computations within deadline, which is novel and different from traditional offloading research, including ones on WSNs. The contributions of this paper are summarized as follows:

- Using *HeaLow*, nodes in WSNs are able to perform heavy-computation processes while satisfying a completion time requirement.
- Unlike related studies which conduct evaluations using simulations only, we implemented *HeaLow* on real devices. Thus, we evaluated *HeaLow* through not only simulations but also several experiments using the devices.
- We consider processing acoustic signals, which can be used for various services and applications in WSNs, as the target task in this paper. Thus, such services and applications are able to utilize *HeaLow* easily.

The remainder of the paper is organized as follows. Firstly, we introduce related work and describe *HeaLow*’s novelties and advantages compared to the related work in Section 2. Secondly, we explain the design of *HeaLow*, the implementation details, and the offloading decision process of *HeaLow* in Section 3. After that, Section 4 explains experiments and simulations, and verifies the performance of *HeaLow*. Finally, Section 5 concludes this paper.

## 2. Related Work

In this section, we introduce studies on cloud-assisted WSNs, task offloading in edge computing, and cooperative computing. In addition, we introduce some of the recent studies of acoustic sensing based applications. After that, we indicate limitations of related work and differences between our research and them.

In general, nodes in WSNs have relatively limited resources, so there have been many studies to overcome the limitation by using powerful resources of cloud servers. Firstly, Wen et al. focused on energy-optimal application execution in their cloud-assisted platform [30]. To reduce the energy consumption of sensor nodes, they formulated strategies to configure the clock frequency and schedule data transmissions optimally. According to their numerical results, a large amount of energy can be saved by offloading task to the cloud clone. Similar to Wen [30], Sinha et al. utilized resources of servers to alleviate the limitations of devices, but they utilized multiple servers together based on their task partitioning algorithm [31]. Their evaluation results show that the proposed partitioning algorithm outperformed existing algorithms. Unlike Wen [30] and Sinha [31], Dattatraya et al. focused on the insufficient storage of sensor nodes [32]. In their system, sensor nodes store sensed data into servers, and the system provides access to authorized users for retrieval of sensor information stored in the servers. Including the above work, many studies indicated that utilizing cloud servers is an effective method to alleviate the resource limitation of sensor nodes. However, utilizing cloud servers is improper for latency-sensitive applications or services required to frequently transmit large-sized data due to the long distances between cloud servers and devices, according to Chandra [16]. Moreover, existing cloud computing models were mainly designed for traditional web applications, so those are not suitable for future Internet applications running on diverse mobile and sensor nodes, including work done by Sheng et al. [19].

In recent years, devices for sensor nodes in WSNs have progressed remarkably, so various processes can be done in sensor nodes without cloud servers. Thus, many researchers such as Varghese et al. focused on edge computing and conducted studies on task offloading which is an important process in edge computing [33]. Firstly, Kyong et al. proposed a method for optimal partition among nodes to minimize the time to search for a pattern of interest in a large data [34]. They conducted a simulation evaluation, and the simulation result shows that the expected time of processing was reduced by partitioning tasks among nodes. Chen et al. designed a distributed computation offloading algorithm and formulated the offloading decision making problem [35]. They conducted numerical studies, and the results of the studies show that their algorithm improves the performance of computation offloading and scales well depending on the number of nodes. Similar to Chen [35], Mao et al. proposed a dynamic computation offloading algorithm which decides the offloading decision, the CPU-cycle frequencies, and the transmission power jointly [36]. The major advantage of this algorithm is that the decisions can be made without requiring other distribution information. The proposed algorithm was evaluated through simulations.

In addition to edge computing, some studies employed the concept of cooperative computing to improve the processing performance and evenly consume nodes’ energy. Sheng et al. proposed a new approach to minimize energy consumption of processing [19]. They evaluated the proposed solution through simulations, and the results of simulations show that the total energy consumption was significantly reduced by distributing workloads among sensor nodes. By using cooperative computing, it is possible for nodes to perform applications which require large computations, such as applications using video or audio data. Thus, cooperative computing can be an effective solution for such applications in WSNs. There are some studies on using cooperative computing to improve the performance of video processing in WSNs. Firstly, Eriksson et al. proposed a solution for optimizing the distribution of tasks among nodes to minimize the time needed to complete the distributed visual analysis in a visual sensor network [37]. Similarly, Long et al. designed a cooperative video processing scheme to maximize the quality of video processing result, and they conducted simulation studies using MATLAB [21].

In addition to video data, audio data are also among the most widely used data types, and there are a lot of studies utilizing audio data in WSNs. Firstly, Mao et al. developed an application which uses Doppler effect to track the user’s motion in real time [38]. Similarly, Zhang et al. designed a system to detect human encounters based on Doppler profiles of sound signals [39]. In addition, Wang et al. proposed a system to perceive surroundings using acoustic reflections [40]. Tung et al. developed a range-free localization technique for room recognition using signal reflections of the surrounding walls and furniture [41]. Recently, there are attempts to adopt acoustic based signals into drone field, which is one of the fastest emerging fields, including Mao’s work for indoor localization [42]. The provided is a recent selection of studies using acoustics, and there are more. While processing acoustic signals, various operations such as FFT and cross correlation are performed essentially. Among the operations, FFT is very frequently used in studies and applications utilizing acoustic signals. Actually, including the above studies, most such studies and applications include the FFT process, and acoustic signals have been one of the primary sources to obtain information in various fields including WSNs, which is well presented in Peng’s work [43]. As acoustic signals can provide information that is not available otherwise, there is an increasing interest in acoustic signals. Acoustic signals are being used in various ways in WSNs: localization, human contact detection, location tagging, guidance, etc. However, most studies using acoustic signals use either specialized devices or smartphones. Many applications including acoustic signal processing require large computations and processing to be completed within a tight deadline. The above requirements are the biggest setbacks to adopt complicated acoustic systems to nodes in WSNs where most nodes only have limited capabilities.

To overcome the aforementioned limitation, we propose the cooperative computing system, *HeaLow*, which enables nodes in WSNs to perform heavy-computation processing with meeting the deadline. Compared to the related work, *HeaLow* has novelties and advantages in several perspectives. First, many related studies focused on processing heavy-computational but delay-tolerant operations. In other words, such studies did not place a lot of emphasis on the low-latency requirement, which means that they cannot be applied to applications requiring both large computations and processing to be completed within a tight deadline. *HeaLow* is designed to perform heavy-computation process while satisfying a completion time requirement, so *HeaLow* can be applied to more diverse applications in various fields. Secondly, including the aforementioned studies, most studies focusing on cooperative computing and offloading in WSNs conducted performance evaluations through simulations without implementation on real devices and experiments using the devices. On the contrary, we implemented *HeaLow* on the real devices and conducted several experiments using the devices to verify the effectiveness of *HeaLow*. Moreover, in this paper, we consider processing acoustic signals, which is one of the most widely used operations in WSNs, as the target task. Thus, many services and applications needing acoustic signal processing are able to utilize *HeaLow* easily.

## 3. The Design of *HeaLow* and Implementation

In this section, we explain the overall design of *HeaLow* first. After that, we explain the implementation details and the techniques used in *HeaLow*. In addition, we give a detailed explanation about the offloading decision process in the availability checker.

It is to be noted that the design and implementation lie in the practical side. Thus, unlike existing studies relevant to offloading or cooperative computing, we focused on devising techniques dealing with practical issues which are difficult to be predicted, not on conducting time scheduling. As *HeaLow* attempts to offload data when the recipient confirms the request, it does not have to deal with complex scheduling issues. Scheduling algorithms may reduce network burdens, but our system cannot afford to try to solve a complex problem with the presence of a tight deadline. For these reasons, we tried to devise techniques to cope with unpredictable situations while conducting heavy-computation and low-latency processing in our system.

### 3.1. Overall Design

**Figure 1:**
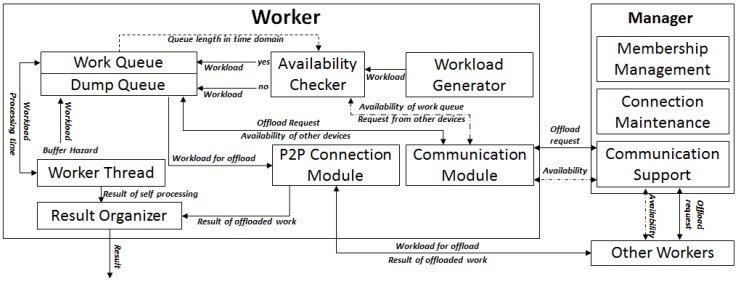
The overall design of *HeaLow*.

Figure 1 shows the layout of the proposed system. First, a manager is a worker device that does slightly more work than the other workers. The manger maintains a socket connection to all the other workers, keeps the membership, and provides a communication bridge among workers. The job of the manager is not heavy, and any device can be a manager. In Figure 1, the left side of the diagram shows operations for the worker. Each square box is a significant entity in *HeaLow*, and the arrows among the squares mean the data or information delivered between the entities. An arrow has one of two descriptions.

The following is how to read the arrow and description notation:

- A one-sided arrow only has one description, which means the data moving in the description are delivered in the direction of the arrow.
- A double-ended arrow has one or two descriptions. If it has one description, the same data are delivered both ways, but the ownership is different. Details are provided with dedicated explanations.
- When a double ended arrow has two descriptions above and below it, the above is a description for data from left to right, and below is the opposite.
- When multiple data flows are connected to an entity, the type of arrow line differentiates the types of data. Details are given with dedicated explanations.

### 3.2. Workflow of HeaLow

In *HeaLow*, the workload generator indicates the module where workloads, i.e., microphone input data, are generated. Through the rest of the manuscript, workload is used with certain amount of ambiguity as we want the concept of our system design to be of use with other systems. However, in this work, we only conducted experiments where a workload is a block of audio input read from the microphone. We have a discrete definition of workload, where a set of microphone input data for one FFT operation is taken as one workload. As mentioned before, most audio related systems use FFT for signal processing, especially when it is also used by other signal processing tools: cross correlation, convolution, auto correlation, etc. It is to be noted that we defined workload as a block of audio input data because FFT is done by blocks. In other studies and systems, this goes straight to a worker thread. This is based on the assumption that the device has enough computational resources. In *HeaLow*, the worker thread has a work queue, because the processing rate can be smaller than the incoming rate. This should be considered in applications for WSNs. When a worker thread has a work queue attached to it, the queue either empties or fills up. If the queue fills up over a certain length, the availability checker denies requests for enqueuing and sends the data to a dump queue. In this manner, the work queue only has an amount that the worker thread can handle.

When a workload is thrown into the dump queue, it needs to either be dropped out or be offloaded somewhere else. The dump queue consistently asks the manager for queue availability of other devices in the membership, where the communication support module of the manager broadcasts the request. The availability checker of another device answers with the amount it can process on top of its own workload, which is delivered back to the requesting device through the communication support module. The dump queue then offloads the number of workloads it is allowed through a peer-to-peer connection to the device. We use the term peer-to-peer connection on the application layer level. In *HeaLow*, a peer-to-peer connection means a connection through which one device sends a data to another device without going through a server device. For instance, the offloading device directly connects to the destination device based on the address given from the server. This avoids a server having to process all traffic. That is, if the devices are connected through Wi-Fi as in our system, if it needs to go through the server’s socket, device A sends the data to the Wi-Fi AP, the AP sends it to the server device, the server device sends it to the AP, and the AP sends it to the destination device. However, if device A directly connects to the destination, transmission only occurs twice. This connection is used again when the result of the offload is returned. The result organizer collects the results directly from the work queue and the offloaded work, and reorders the results for the application. Some of the missing explanation for implementation details are given in Section 3.3, along with the design choices for them.

### 3.3. Implementation Details

In this subsection, we explain implementation details as well as the issues that arose. We have implemented various algorithms and techniques to optimize the performance of *HeaLow*, and these are explained in this section. *HeaLow* was implemented on Android. The descriptions given in this section are based on Figure 1 if no other specification is provided.

#### 3.3.1. Buffered Load Management

By buffering workloads throughout *HeaLow*, we improved the efficiency of *HeaLow*. Especially for networks, buffered load delivery maximizes the network usage. To make use of the buffers, the availability checker returns the number of workloads it can process when other devices request for offload. On the other hand, true or false is returned for its own work which has not been buffered yet. In *HeaLow*, the availability indicates the maximum queue length divided by the processing rate.

Network transmissions are made with buffers as well. The transmissions are made in the transmission thread with a buffer that other threads can add messages to. In this manner, when the network is available, a device can send multiple messages in the queue that have the same destination with a single access. One prolonged communication has better network utilization than two half-length communications. We named this method, sending multiple messages to the same destination as one, *buffered message method*. One may concern this can cause one device to overtake all network bandwidth, but the only possible source of the unfairness is workload, which is sent when a reply to the request is received.

#### 3.3.2. Work Queue Management

The most important component of a worker in *HeaLow* is the work queue. As implied by its name, a workload at the head of the work queue is processed by the worker thread. Of course, a workload that has passed its deadline is consistently removed from the queue, and this event is considered as a failure. The availability checker determines if a workload can be processed by the worker thread. The amount that cannot be processed is thrown into the dump queue, and workloads in the dump queue are sent to other devices or considered as failure. The queue length of *HeaLow* is perceived in time domain, as deadline is one of the key design issues of *HeaLow*. The worker thread reports the processing time of the last workload to the work queue, where the amount of work in the work queue is divided by the processing rate to get the queue length in time domain. The queue length is then compared to the new workload’s deadline. If the queue length is not smaller than the deadline, the workload cannot be added to the queue. The detailed explanation about the offloading decision process in the availability checker is given in Section 3.4.

**Figure 2:**
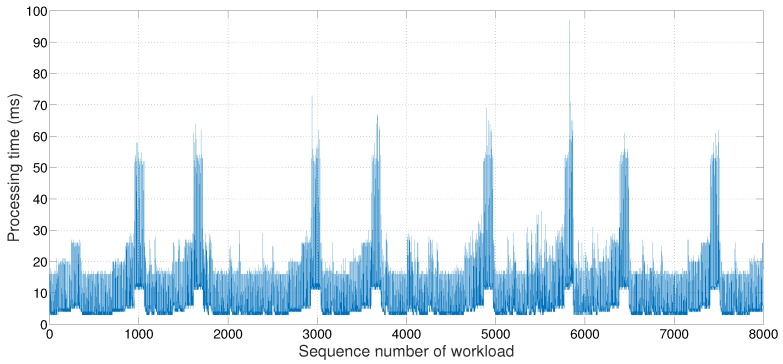
The workload processing time in the worker thread.

The above method is logical and straightforward, but the processing time brings a practical issue. As shown in Figure 2, the processing time often slows down to an order of magnitude for a short period. Although we could not pinpoint the cause of the delay, our best guess is that the android OS is not performing operations for only *HeaLow*, and this created a competition for available resources. This results in a phenomenon we named buffer hazard, and we implemented an additional technique to deal with this phenomenon. A buffer hazard occurs when the processing time slows down rapidly, multiplying the perceived queue length, rejecting all incoming workload for a period of time while unable to finish the workload already in the work queue either. When a buffer hazard occurs, we can anticipate its processing rate will be slow for a short period. Therefore, when it happens, the device enters the hazard state and offloads a portion of incoming workload without checking availability until the queue empties and exits the hazard state. We named this method *hazard state management*.

If workloads are offloaded without the *hazard state management* approach, the queue length is perceived to be available to accept more workloads. This brings an extremely unstable behavior to the queue, as workloads accepted will create another hazard state. The workloads that are sent to the dump queue will have a scrambled order. Although all queues are consistently sorted, accesses to the queue may create an unpredicted behavior. The *hazard state management* effectively handles the sudden increase in processing time, especially when the processing time increases over a few workloads.

#### 3.3.3. Communication Module and Delayed Response

There are two different modules for communication in *HeaLow*. The communication module is connected to the manager, and the peer-to-peer connection module is not connected anywhere. If all messages are sent through the manager that keeps connection for all its members, the workers do not need to have any information of other members. In this manner, the number of connection to keep all devices connected is minimized. This is especially convenient when sending a message to all members, for instance when sending an offload request message. However, this doubles the number of transmissions for each message delivery, as the manager has to send the message again. This is normally not a critical issue, but, when the message size gets bigger with the offloading workload and results, the doubled network usage creates a serious network bottleneck. To avoid such bottleneck, large-size messages that include workloads or process results are sent directly to the destination. Since every offload starts with a request and an answer, the answer message includes the network address information for the requester to create a socket through the peer-to-peer connection module. This method effectively halves the network usage unless the manager acts as the wireless infrastructure.

Again, as shown in Figure 2, the processing rate slows down for a period of time. The amount of workload offloaded proportionally increases in that period, using network bandwidth accordingly. Each workload is processed in milliseconds range, and immediately returning the result doubles the network usage. Since the competition and the congestion increase along with increased network usage, total effective network bandwidth drops below half when the usage is doubled. Therefore, if the offload process is complete, the receiving device monitors the time left until the deadline and holds on to the result if more than twice the network transmission time is left. This simple technique improves the overall performance effectively. We named this method *delayed response*.

### 3.4. Offloading Decision Process in Availability Checker

In *HeaLow*, a device (a worker) may process a workload by itself and finish the processing by the workload’s deadline. Otherwise, the workload is offloaded to one of the device’s neighbor devices, and the selected neighbor performs the processing for the workload owner. As explained briefly in the previous subsection, the availability checker decides whether workloads are offloaded or not. The proper decision making is important in *HeaLow*, so various factors should be comprehensively considered for appropriate decisions. For this reason, in this subsection, we give the detailed explanation about the offloading decision process in the availability checker.

#### 3.4.1. Fundamental Situation Assumed for Describing Offloading Decision Process

We assume a situation where there are a main device and n−1 neighbor devices. One neighbor device takes the role of the manager, and all devices are within the same network. $buffer_{t}^{i}$ indicates the amount of buffered workloads in the work queue of the *i*-th device at time *t*, and the default unit for task is one workload. $wl_{n}^{i}$ means the *i*-th device’s *n*-th workload. $dl\_wl_{n}^{i}$ and $si\_wl_{n}^{i}$ mean the deadline time and the data size of $wl_{n}^{i}$ respectively. A device conducts sensing and sampling, so a workload consists of samples, and $ns\_wl_{n}^{i}$ is the number of samples in $wl_{n}^{i}$. In *HeaLow*, xlogx is the amount of computation for processing the workload composed of *x* samples. $r_{proc}^{i}$ and $r_{tran}^{i}$ are the processing rate and the transmission rate of *i*-th device. In *HeaLow*, $buffer_{t}$, $r_{proc}$, and $r_{tran}$ are shared through the manager as explained before. Table 1 lists notations and variables used for *HeaLow*.

In this situation, there are two major cases to be considered. The first case is when a device processes the device’s workload, and the other case is when the device is requested to process another device’s workload.

#### 3.4.2. The Case When a Device Processes the Device’s Workload

In *HeaLow*, a device prefers to process the device’s workload by itself than to request one of neighbors to process the data. However, the device may not be able to finish the processing by the workload’s deadline for various reasons, such as low computing power. In such situations, the device should offload the workload to a neighbor device and request the neighbor to process the workload. *HeaLow* makes best effort to finish the processing by the deadline. If it cannot, it offloads or removes the workload. Thus, before starting the processing, the device should be able to predict whether the device can finish the processing by the deadline or not.

When a new workload is created, the device decides whether the workload is inserted into the work queue or offloaded to another neighbor. At this moment, suppose that there are *x* workloads in the work queue. In this situation, the device checks whether the device is able to finish processing the new workload by the deadline or not, based on Inequality (1) when the device is the *i*-th device. In Inequality (1), $ft\_bw_{x}^{i}$ means the time to finish processing *x*-th buffered workload in *i*-th device’s work queue, and $dl\_wl_{new}^{i}$ indicates the deadline time of the new workload, $wl_{new}^{i}$. As we explained before, $ns\_wl_{new}^{i}\log ns\_wl_{new}^{i}$ is the amount of computation for processing $wl_{new}^{i}$.

(1) $$ft\_bw_{x}^{i}+\frac{ns\_wl_{new}^{i}\log ns\_wl_{new}^{i}}{r_{proc}^{i}}\leq dl\_wl_{new}^{i}.$$

However, in practical situations, the operation of processing buffered workloads can be delayed unexpectedly, as shown in Figure 2. If such unexpected events occur so that the device cannot finish processing the new workload by the deadline, the workload should be offloaded to another neighbor, *j*, and the processing result of the workload should be returned to the device by the deadline. Thus, in addition to Inequality (1), another condition, Inequality (2), should also be considered.

(2) $$ft\_bw_{x}^{i}+\frac{si\_wl_{new}^{i}}{r_{trans}^{i}}+\frac{ns\_wl_{new}^{i}\log ns\_wl_{new}^{i}}{r_{proc}^{j}}+\frac{si\_pw_{new}^{i}}{r_{trans}^{i}}\leq dl\_wl_{new}^{i}.$$

In Inequality (2), $pw_{new}^{i}$ is the processed workload of $wl_{new}^{i}$, and $si\_pw_{new}^{i}$ means the size of $pw_{new}^{i}$. We named this approach *buffer length management* in *HeaLow*.

If Inequalities (1) and (2) are valid, the device finally puts the new workload into the work queue to process the workload by itself, and then the number of buffered workloads becomes x+1. After that, the time to finish processing all the buffered workloads is reset as follows:

(3) $$ft\_bw_{x+1}^{i}=ft\_bw_{x}^{i}+\frac{ns\_wl_{new}^{i}\log ns\_wl_{new}^{i}}{r_{proc}^{i}}.$$

If either Inequality (1) or Inequality (2) is not valid, the device offloads the workload to one of neighbors.

#### 3.4.3. The Case When a Device Is Requested to Process Another Device’s Workload

The second case is when a device is requested to process the workload of one of neighbors. As explained before, when one of the neighbors needs to offload some of its workloads, the neighbor broadcasts this situation through the manager with the deadline information, dl. After the device receives a broadcast message, the device sends a reply message to the neighbor. The reply message contains information about $am_{comp}^{i}$ which means the amount of computation that the device, *i*, is able to process by the deadline. $am_{comp}^{i}$ is calculated as follows:

(4) $$am_{comp}^{i}=\left(dl-ft\_bw^{i}\right)/r_{proc}^{i}.$$

After the neighbor receives the reply message, the neighbor sends workloads to the device, *i*, according to $am_{comp}^{i}$. After that, the device receives the workloads, puts the workloads into the work queue, and resets $ft\_bw_{x}^{i}$, as explained in Equation (3).

## 4. Performance Evaluation

To evaluate *HeaLow*, we implemented *HeaLow* on real devices and conducted experiments using the devices. In addition, to analyze *HeaLow* from various perspectives, we conducted simulations using the simulator we designed. We describe details of the experiments and analyze the result of the experiments in Section 4.1. After that, we give an explanation about simulations and interpret the result of the simulations in Section 4.2.

### 4.1. Experiments

We implemented *HeaLow* on off-the-shelf devices named *Samsung Galaxy Nexus* with Android OS. Using the devices, we conducted experiments to analyze effectiveness of techniques used for *HeaLow*.

#### 4.1.1. Analysis on Effectiveness of Techniques Used for *HeaLow*

**Figure 3:**
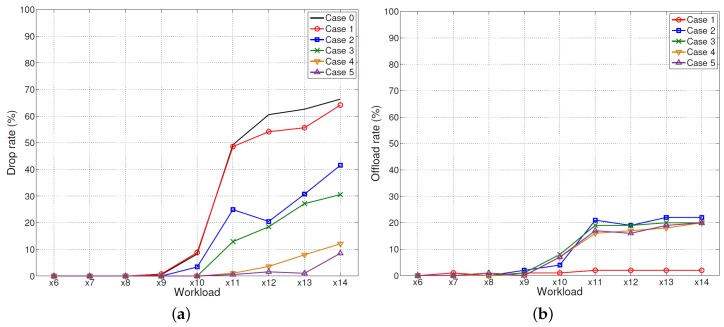
The effectivenesses of the techniques used for *HeaLow*: (**a**) the drop rate of each case; and (**b**) the offload rate of each case.

**Figure 4:**
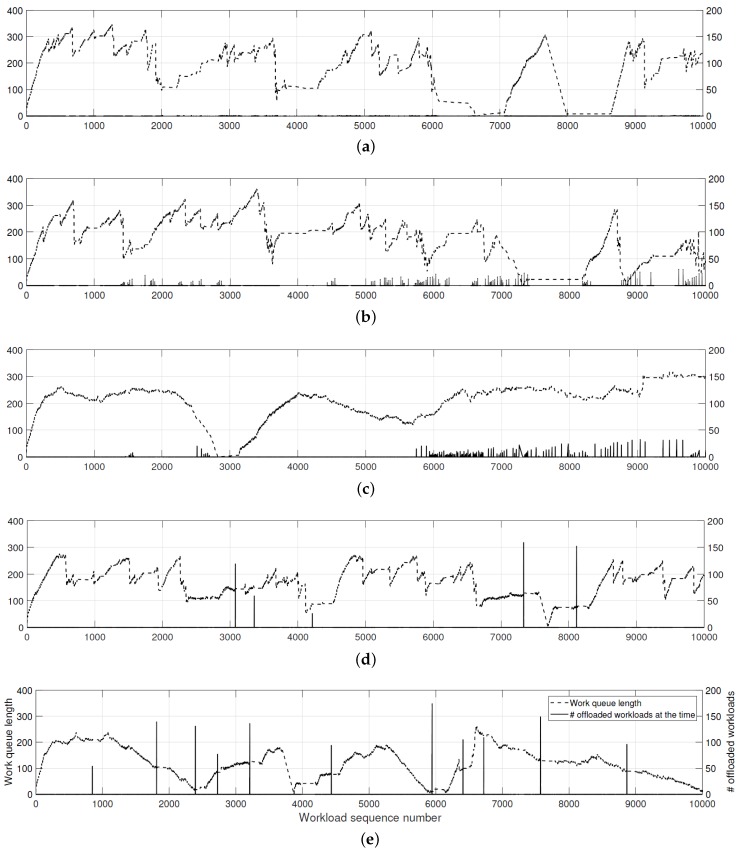
The queue length and the number of offloaded workloads with respect to workload sequence number: (**a**) Case 1; (**b**) Case 2; (**c**) Case 3; (**d**) Case 4; and (**e**) Case 5.

As explained in Section 3.3, we have implemented various algorithms and techniques to optimize the performance of *HeaLow*. In addition, we conducted some experiments to analyze the effectiveness of the techniques, as presented in this section. Figure 3 shows the drop rate and the offload rate of *HeaLow* with and without various optimization techniques we have adopted. The “×” notation in x-axis indicates the amount of workload generated compared to a basic state. In the experiments, we used smartphones, so the devices had powerful computation powers. However, it is to be noted that the performance of smartphones degrades quickly over time, which is why we tested intermittently with 15-min interval for more reliable results. The methodology used to obtain Figure 4 is as follows. When a workload is being offloaded, the system counts the number of offloaded workloads as it returns to the device. The queue length is recorded when each of workload processing is finished. Therefore, the peaks shown in Figure 4d,e may seem out of place as the peaks are recorded when the packets are returned to the original device. As we implemented delayed response, this causes the peaks to be a few hundred milliseconds to seconds later than when they are offloaded.

First, each case is as follows. Case 0 is for a single device processing all data by itself, which means that *HeaLow* was not applied, and Case 0 stands for the case of current WSNs. In Case 0, there is no other device to offload to, so Case 0 is not shown in the offloaded ratio graph. In Case 1, we applied no optimization method as a base case. In Case 2, we added *buffered message method* which is the biggest improvement for the performance. *Buffered message method*, as explained in Section 3.3.1, is a method of using the network bandwidth with a message buffer, sending multiple messages together instead of sending one by one. In Case 3, *buffer length management* and *hazard state management* have been added on top of Case 2, because *buffer length management* is a supportive technique for other following techniques. *Buffer length management* mentioned in Section 3.4.2 is a method of maintaining the length of the buffer to consider the network delay and the current processing power. *Hazard state management*, as explained in Section 3.3.2, is a method to manage the device in two states, stabilizing the behavior of offloading. *Delayed response* explained in Section 3.3.3 is a method for the receiving device to monitor the time left until the deadline and hold on to the result if more than twice the network transmission time is left. Case 4 has *delayed response* and *buffer length management* on top of Case 2. Case 5 has all four methods implemented.

In Case 0, which is without *HeaLow*, there are a lot of failed workloads because it is difficult for a single node to perform heavy-computation and low-latency processing by itself. In Case 1, no matter how many workloads are failing, offloading does not exceed 2%. Since each device is blind to other devices’ status, each offloading needs a permission from the target device. Sending each packet after permission means each workload requires three-way packet exchange (request, response, and offloading), so the performance is unacceptably low. In Case 2, the amount of offloading increases to 22% as dropping packets increase. As a result, the sum of amount being dropped and offloaded is close to the amount being dropped in Case 1. This is expected as network utilization increases significantly with *buffered message method*. In Case 3, the amount being offloaded does not increase compared to Case 2. Rather, it decreases slightly, while the amount being dropped decreases significantly. At first glance, it may not be logical to have the same amount offloaded and have fewer failures. When a buffer hazard occurs, without *hazard state management*, a large portion of workload already in the work queue drops. In such situation, buffer intermittently rejects all workload for a short period. The network cannot handle such request, hence higher drop rate. By stabilizing the queue preemptively, the device can process comparably durable load for longer interval. Case 4 may also seem odd. Offloaded amount can also be dropped in either end of offloading process when deadline has passed. This means that a severe network bottleneck can cause packet drop even more severely. Hence, better network utilization can have a better success rate with the same amount of offload.

Especially in ×10 workload case, we can clearly see that Case 3 makes more offloading than Case 2 with preemptive queue stabilization. To avoid any confusion, × notation means the number of audio streams that a device can process. The most important issue is that, with Cases 3, 4, and 5 on top of Case 2, the success rate increases without having more data offloaded. Since offloading consumes the computing resource of other devices and the network bandwidth, improving performance without network usage is always desirable. Through this experiment using real devices, we confirmed that *HeaLow* with various techniques enables nodes to perform heavy-computation processes while satisfying a completion time requirement.

#### 4.1.2. Analysis on Queue Length and Offloading

To better understand the behaviors with our techniques, we recorded the queue length and the number of offloaded workloads, as shown in Figure 4. The cases in the figure are explained in the previous section. This experiment was carried out with a pair of smartphones, with ×10 workloads. The performance of this experiment may not match the result in the previous section, because we added file writing code to analyze each processed workload. The added write process was a significant burden to the devices. Therefore, the perceived processing power of the device is relatively limited.

From Case 1 to Case 2, the added technique, *buffered message method*, allows *HeaLow* to offload much more, improving the overall performance. In Case 3, the average queue length stabilizes, having less deviation. With *buffer length management* and *hazard state management* techniques, the device manages the queue length to stick around to the desirable length, so the deviation for the queue length is minimized. In Cases 4 and 5, the queue length is shorter, and offloading seems to last shorter than other cases. *Delayed response* method alleviates packet contention and allows the device to offload more than five times within the same period of time. In addition, while the recipient device awaits for the deadline of the offloaded workload, more offloaded workload is processed, causing a chunk of workloads to be returned at the same time. As a result, shortened offloading time helps with the overall workload management.

With the result shown in Figure 4, we can see that the most important factor to consider in offloading is the network congestion. Therefore, the performance improves significantly with *delayed response*. In that sense, *hazard state management* is an indirect method of considering the unpredictable network, by forcing the device to evenly access the network over a period of time instead of abruptly demanding network resource when needed.

### 4.2. Simulations

In this section, the simulations conducted with the simulator we designed are reported. The simulator was written in python script, with all actual code ported in the simulated device. The localization simulation explained in Section 4.2.3 was conducted with a different script, also written in python script.

#### 4.2.1. Simulation on Effectiveness of *HeaLow* in WSNs

**Figure 5:**
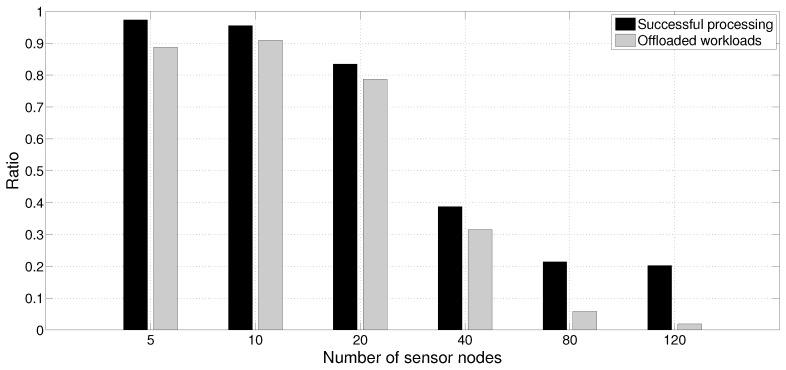
The successfully processed and offloaded amount in a sensor network environment.

In our first simulation, we set up an environment where there is a high-end smartphone offloading data from multiple sensor sources using *HeaLow*. In this scenario, we examine the applicability of our system to WSN where a powerful device is nearby. An example scenario was to have microphone based sensor nodes exploiting a user’s smartphone when one is nearby. In this way, the user can utilize more microphone inputs when available, and inputs to the sensor nodes can be used with deeper understanding. Figure 5 shows the average ratio of successful processing and offloaded amounts. We simulated an environment assuming a commercial high-end smartphone that can process up to ×25. This number is based on the data in Section 4.1.1 where a low-end smartphone has ×8 to ×10 processing power. Each sensor node can only process 20% of its input. With our system, a node will try to offload the other 80% and more, although these may fail to meet deadline due to the limited capacity of the high-power device and the network bandwidth limitation. Throughout the simulation, only one high-end smartphone was placed. There was no low-end smartphone. In addition, each sensor node generates ×1 workloads.

With any given point of our simulations, each sensor node will try to offload the same amount, which is more than 80%. As shown in the figure, when there are five sensor nodes, a node offloads roughly 90% of its workload and fails to process less than 3% of the workload. With 10 nodes, a similar result is shown with more drop rate, and we found that this is due to the network congestion. Our system asks if the offloaded device have a room for more task, and offloads only when there is. In this case, the only device that can do offloaded work is the smartphone, and the amount of each sensor’s offloaded workload decreases when the smartphone does not reply. This is highly likely due to network bottleneck. The success ratio visibly drops from 20 nodes, and the offloaded amount drops significantly from 80 nodes. However, it has to be noted that the amount of offloaded data exceeds the amount of data traffic in average WSN. If we lower the sampling rate, the network bottleneck is expected to be mitigated as well. In summary, when using *HeaLow*, a smartphone device is sufficient to enable up to at least 20 sensor nodes that are not capable of processing an average acoustic sensor input.

#### 4.2.2. Analysis on Processing Capability of Nodes Using *HeaLow*

In our second simulation, we set up an environment where multiple smartphones process intense amount of workload. The concept is that higher resolution brings finer grained information. Hence, it is better to do as much work as possible if no other issue is considered. More detailed explanation on how the precision and accuracy is affected is discussed below.

**Figure 6:**
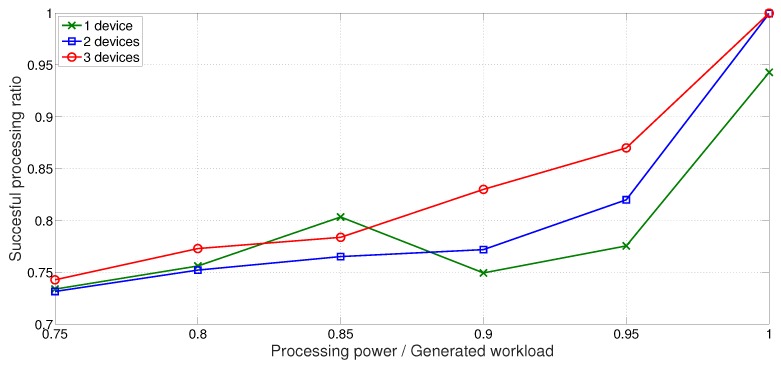
Smartphone processing simulation.

As explained before, a buffer hazard incident occurs because the processing rate intermittently drops down to a tenth of average processing time. Therefore, even when a device is capable of handling the acoustic sensor input, workloads are dropped unless the capability is a few times greater than the input data. Figure 6 shows such result. Y-axis shows the successful processing ratio, and x-axis is the processing power relative to the generated workload. For example, if the processing rate is 0.5, the device is capable of processing only half of its generated workload. In a single device scenario, 5% of workloads were dropped even when the value of processing power over generated workload is 1. As the number of devices grows, the amount of processing power and generated workload grows proportionally. Therefore, the scenario of three devices completing higher ratio of workloads when there is sufficient computing power shows that *HeaLow* stabilizes performance when there is enough processing power in the entire system.

However, when any of the devices is not capable of extra workload, as shown in (0.75, 0.85) range, *HeaLow* cannot give much benefit. In addition, it is worth noting that the success ratio hikes as × gets close to 1. This is because the cost of queue management significantly drops when the device can process as much as it generates.

#### 4.2.3. Analysis on Quality of Service Improved by Using *HeaLow* in WSNs

In this section, we conducted an acoustic-related simulation. We argued that higher resolution brings finer grained results. That is, if a device using *HeaLow* can process the same acoustic signal with larger FFT indices, the result will be more accurate. First, we assume a smartphone device where the speaker and the microphone is separated 15 cm. After that, we implemented a chirp signal, and detect the chirp peak after cross correlation. Such implementation details are a naïve implementation of an acoustic ranging application in Jung’s work [22]. In other words, the sending sensor device offloads the data with 8000 Hz sampling rate. Instead of running FFT with 8000 Hz, the receiving device runs *N* times larger FFT, the coefficient of which is denoted as ×*N*.

There are two common methods of acoustic localization, one being a dead reckoning by tracking the velocity of the device using Doppler effect, the other being an exact location estimation using chirp detection. In this section, the target application we use is the latter, because we wanted to show the result that is independent of filtering algorithms that must accompany the former. In a chirp method, the distance between two devices is estimated using the time of arrival of the signals. This means that the accuracy and precision of the time of arrival will decide how accurate the distance is, and hence decides the accuracy of the location estimation. The exact method of localization after distance was obtained was MDS (Multi Dimensional Scaling).

**Figure 7:**
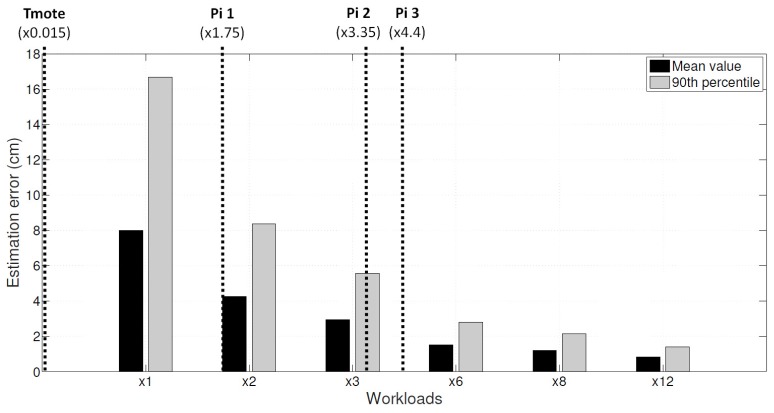
Localization error varying on the amount of workloads.

Figure 7 shows that the result improves even with the same data input. The result of chirp detection relies heavily on the accuracy and granularity of the peak detection. The peak detection uses cross correlation, which is composed of three FFT operations. The precision of the peak detection improves with higher resolution input, so the result improves as well. This result means that the use of *HeaLow* can improve the quality of applications or services operated in WSNs.

*TMote Sky* (*TMote Sky* was released in 2005) is the wireless sensor module, and the *Raspberry Pi* (*Raspberry Pi 1*, *Pi 2*, and *Pi 3* were released in 2012, 2015, and 2016 respectively) is a series of small single-board computers. According to Shin [44], performances of *TMote Sky* and *Raspberry Pi 2* were compared. Similarly, benchmarks available online in [45,46] run code for single precision FFT of size 1024K on *Raspberry Pi 2*, *Raspberry Pi 3*, and *Nexus 7*. In addition, Sysbench compared performances of *Raspberry Pi 1*, *Raspberry Pi 2*, and *Raspberry Pi 3*, which is available online in [47]. Geekbench also compared performances of *Nexus 7* (*Nexus 7* was released in 2012) and *Galaxy Nexus* (*Galaxy Nexus* was released in 2011), which is available online in [48]. Based on the above benchmark results, we roughly surmises that *TMote Sky*, *Raspberry Pi 1*, *Raspberry Pi 2*, and *Raspberry Pi 3* have 0.30%, 35%, 67%, and 88% of computational power compared to *Galaxy Nexus*, respectively. In other words, they can process about ×0.015, ×1.75, ×3.35, and ×4.40 workloads, respectively, as shown in Figure 7. This means *Raspberry Pi 1* can only have highly erroneous results while a *TMote Sky* node cannot provide acoustic capability at all. However, *TMote Sky* nodes have 250 kbps communication capability. This translates to 125,000 samples per second, allowing *TMote Sky* nodes to have ×3 workload capability when using *HeaLow*. Of course, the maximum network throughput is not achieved in practice, but we can still safely expect ×1 workload processing by using *TMote Sky* nodes with *HeaLow*.

Note that these specifications are only a rough estimation of the computing power of the devices, and not exact. Therefore, these values can be tens of magnitude different from the actual computing power, but for the purpose they serve, they are sufficient.

## 5. Conclusions

Compared to traditional nodes in WSNs, today’s sensor nodes have more powerful resources, so the nodes are able to carry out more complicated operations and more diverse applications can be applied to WSNs. Among the applications, many applications deal with audio data because audio data are among the most widely used data types, and sensor nodes are able to utilize microphones easily. However, most applications require large computations and processing to be completed within a tight deadline, so it is difficult for a single node to run relatively heavy applications by itself. To overcome this limitation, in this paper, we proposed the cooperative computing system, *HeaLow*, for heavy-computation and low-latency processing in WSNs. Unlike related studies which evaluate their own systems through simulations only, we implemented *HeaLow* on real devices. Thus, we conducted experiments using real devices as well as simulations, and the results showed that nodes using *HeaLow* in WSNs are able to run applications which require heavy operations with low-latency. We consider processing acoustic signals, which can be used for various services and applications in WSNs, as the target task in this paper. Thus, such services and applications are able to utilize *HeaLow* easily. However, since *HeaLow* was not designed to be specialized in specific applications, *HeaLow* can be used for any application.

We have several directions for future work. Since *HeaLow* attempts to offload data when the recipient confirms the request, the duty cycling of WSNs is not a problem to run our system. However, the performance of the proposed system might be degraded due to neighbors in sleep state. Thus, we will analyze the performance of our system applied to WSNs with duty cycling. In addition, we plan to theoretically analyze whether *HeaLow* can be generalized to deal with representative task assignment problems. In addition, we would like to implement the proposed system on more various platforms and evaluate the system in a variety of situations.

## Figures and Tables

**Table 1:** Notations and variables used for *HeaLow*.

Notation/Variable (Unit)	Description
$wl_{n}^{i}$	*i*-th device’s *n*-th workload
$pw_{n}^{i}$	The processed workload of $wl_{n}^{i}$
$dl\_wl_{n}^{i}$	The deadline time of $wl_{n}^{i}$
$ns\_wl_{n}^{i}$	The number of samples in $wl_{n}^{i}$
$ft\_bw_{x}^{i}$	The finish time of processing *x*-th buffered workload in *i*-th device’s work queue
$buffer_{t}^{i}$	The amount of buffered workload at time *t* (KB)
$si\_wl_{n}^{i}$	The data size of $wl_{n}^{i}$ (KB)
$si\_pw_{n}^{i}$	The data size of $pw_{n}^{i}$ (KB)
$r_{tran}^{i}$	The transmission rate of *i*-th device (KB/s)
$r_{proc}^{i}$	The processing rate of *i*-th device (The number of samples per second)

## Abbreviations

The following abbreviations are used in this manuscript:

WSN	Wireless Sensor Network
IoT	Internet of Things
MEMS	Micro Electro Mechanical Systems
FFT	Fast Fourier Transform
